# Whole health implementation lessons from the Veterans Health Administration, United States of America 

**DOI:** 10.2471/BLT.25.293503

**Published:** 2025-10-14

**Authors:** Alex H Krist, Tracy Gaudet

**Affiliations:** a Department of Family Medicine and Population Health, Virginia Commonwealth University, 830 East Main Street, room 631, 23219 Richmond Virginia, United States of America (USA).; b Cornerstone Collaboration for Societal Change, Winkelman, USA.

Health, a fundamental human right, is more than the mere absence of disease: it includes physical, mental and social well-being.^1^ In the United States of America, the population faces a growing health crisis. Life expectancy is declining, maternal and infant mortality rates are rising, and chronic diseases are becoming more prevalent.^2^^,^^3^ The country's clinician-centric health-care system is disease-focused, reductionist and transactional and not designed to engage or equip people to address their self-care or promote prevention and well-being. Socioeconomic, structural and environmental factors further contribute to and reinforce poor health.^2^

A new approach to health, called whole health, is gaining popularity, with a prominent example being the Veterans Health Administration. Whole health re-envisions what it means to be healthy and how to help people achieve it, and it is fundamentally different to the way the United States currently approaches health care. Based on a review of 12 whole health exemplars, the National Academies of Sciences, Engineering and Medicine identified the five foundational elements for effective whole health systems as being (i) people-centred; (ii) comprehensive and holistic; (iii) upstream-focused by addressing health behaviours, and social and structural drivers of health; (iv) equitable and accountable to ensure health for all; and (v) dedicated to ensuring the well-being of the team providing care.^4^

The whole health approaches identified in the report varied in design, scope, operation of foundational elements and people served, highlighting that the whole health approach can be adapted to work in a range of settings and needs. Examples from the United States included health systems approaches, a community health centre, condition-specific clinics, insurance-based approaches and a state-based payment model. International approaches included community and/or regional approaches in Germany, New Zealand (Canterbury) and Spain (Basque Country); a national approach (Costa Rica); and a policy approach (Australia).^4^^–^^11^ Collectively, evidence demonstrated that whole health improved patient care experience and quality measures; increased access to care and reduced emergency department use and hospitalizations; improved management of chronic pain, mental health, traumatic brain injury and healthy ageing; reduced maternal and infant mortality; improved health equity; and reduced health-care expenditures.^4^

**Fig. 1:**
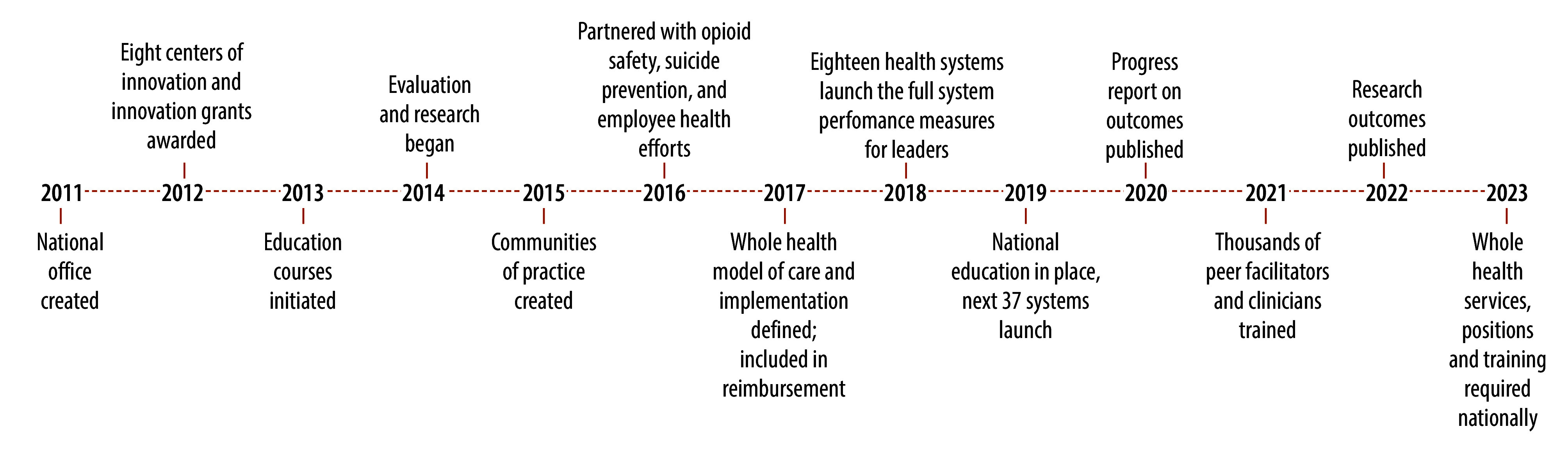
Timeline of system-level changes by the Veterans Health Administration to advance their whole health approach, United States of America

Note: The Veterans Health Administration took an iterative, system-wide approach to transforming its health-care delivery model towards whole health. Beginning in 2011 with the creation of a national office, the administration progressively implemented foundational changes across research, education, reimbursement, leadership, evaluation and employee well-being. Each step informed and reinforced the next, enabling continuous innovation and adaptation. Over time, these coordinated efforts fostered a cultural shift and established a whole health approach including services, training and infrastructure that serves as a national standard.

The National Academies’ report identified the Veterans Health Administration as a leader in providing whole health care. This health-care system started delivering whole health services in 2011, with 200 innovation projects and eight innovation centres to test ideas and operationalize approaches.^12^^,^^13^ This phase was followed by the creation of 25 design sites to refine the approach, then full-scale deployment at 18 flagship health-care facilities. The programme had broad uptake by Veterans, and the administration is now expanding the whole health-care programme across its entire system.^13^ As of 2023, more than 1.8 million Veterans (more than one third of those receiving care through this health system) participated in whole health services (Fig. 1).^14^

The Veterans Health Administration’s whole health-care programme includes three components. First, the pathway, where peers help the Veterans explore their mission, aspiration and purpose and develop a personal health plan. Second, clinical care, which integrates allopathic and complementary integrative health to support the Veterans’ personal health plan. Finally, well-being programmes, with health coaching, self-care and skill-building groups that equip the Veterans with improving their health.^15^ This approach has led to improvements for patients (better chronic pain and mental health management, enhanced well-being, increased self-care, adherence with preventive and chronic care); employees (reduced burnout, greater professional satisfaction); and the health system (higher employee retention, improved satisfaction scores and quality measures, lower pharmacy costs).^4^^,^^15^^,^^16^

This whole health programme represents a transformation of the health system, shifting from a model focused primarily on diagnosing and treating disease to one that empowers and equips people to take charge of their well-being. However, such system change will only be effective if patients want what it offers. Data from the programme reveal that Veterans prefer whole health care, particularly those struggling with aspects of their lives.^4^ If the whole health model works for this vulnerable population, it may also work more broadly. While this health-care system is an ideal setting for transformation, being a nonprofit organization that serves the needs of Veterans, lessons can be learnt from its experiences and from other whole health exemplars, on how to scale and spread whole health. 

To drive system transformation, this health-care system underwent a fundamental cultural transformation to its organizing principles, approach and locus of control for care. The organizing principle for whole health requires a shift from a problem-based to a purpose-driven focus. The power of purpose becomes the driver of self-care and healthy behaviours. The approach for whole health reframes the concept of care from medicalizing to humanizing. Rather than merely focusing on medical priority, treatment plans and guideline-directed care, attention is first directed to addressing the person, their life circumstances and individual values. Doing so builds trust and creates alignment between care plans and what people want and need. The locus of control for whole health care needs to shift from the health system to the community, placing trust on people and communities to know what is best for them. The centre of care moves from the clinic to where people live, work, learn and play through community, peer-based, well-being programmes, building on the local culture, values and resources of the community.

The Veterans Health Administration used several strategies that were essential to success and can be adopted by other systems using a systems approach. Changes were required in the model of health care and in the reimbursement system, performance evaluations, approach to research and outcomes, and team training on care delivery. These components were addressed simultaneously rather than individually or sequentially. Bottom-up grassroots strategies were also essential. For example, identifying clinical and training champions within each facility; training veteran peers to facilitate whole health; recognizing and supporting innovation through individual grants to design sites and facilities to be centres of innovation; and supporting the whole health care team with implementation guides, toolkits and mentoring. The process created a network of champions to share whole health experiences and resources while creating a sense of ownership to motivate others to be part of the transformation. Top-down strategies included changes to reimbursement and performance evaluations, identification and removal of systems barriers, and prioritizing, resourcing and incentivizing whole health activities. Efforts were supported by authentic partnerships working towards a shared vision of transformation. This process created a learning health system that shared lessons learnt, challenges, successes and best practices.^12^^,^^17^

Despite successes, there are common challenges across health systems with scaling and spreading whole health. As the concept of whole health becomes more popularized, the term is being misappropriated. True whole health care requires attention to all the foundational elements. Comprehensive and holistic care goes beyond including complementary and integrative medicine: it focuses on the physical, behavioural, spiritual and socioeconomic needs of the person. The Veterans Health Administration demonstrated that Veterans receiving full whole health care had greater and more sustained benefits than those only receiving complementary and integrative health in addition to usual care.^16^ The design of whole health care will vary across communities. Authentic engagement to tailor a whole health system’s design to meet their community’s needs is time- and resource-intensive. Southcentral Foundation’s Nuka System of Care created a customer-driven overhaul of its system, and the Alaska Native people it served were considered customer-owners who defined the vision, mission and focus of care. The change fostered an environment for creativity, innovation and continuous quality improvement.^6^

Addressing upstream factors, specifically the social drivers of health, is essential for success. These drivers influence health more than health care itself but are much more difficult to address. The Veterans Health Administration developed tools and services to support Veterans with social needs, which have been integrated into the whole health programme.^18^ Ensuring equity and accountability is also challenging, because it requires defining the community that the system serves. The administration is responsible for Veterans, so the community they serve is clearly defined. The Costa Rican government took a national approach, creating the *Equipo Básico de Atención Integral de Salud* system and making it responsible for every citizen. Since its implementation in 1995, more than 90% of Costa Ricans have primary health-care access, the foundation of their health system.^19^ This accountability is an essential function for whole health systems, as those not accounted for by any health system are likely the ones most in need of care.

In its report, the National Academies issued six overarching recommendations to guide health systems in scaling and spreading whole health. While directed to national policies, the concepts of the recommendations are applicable internationally as well. The report outlined the essential infrastructure required for whole health systems, including health informatics, workforce training and well-being, measurements for learning, accountability and financing. The report also emphasized the need to evaluate both implementation processes and outcomes to inform continuous improvement, and called for the establishment of a national health innovation centre to advance policies and payment models that support whole health. The report called for the Veterans Health Administration to continue upscaling within its system and spreading its whole health programme to other systems so that every veteran can access this type of care. While the current United States budget increased funding for the Veterans Health Administration, broader efforts to improve government efficiency have led to workforce reductions, complicating efforts to fill key positions needed to deliver whole health care. As the United States government is further closing and consolidating government agencies, the needed whole health innovation centre is unlikely to be established. Given the programme’s success, sustained support for the whole health initiative and full implementation of the National Academies’ recommendations is critical to realize health for all.

Similarly, while some health systems have made progress in whole health care, more change is needed. Individual clinicians, practices and communities can make meaningful action to advance whole health, even without broader support.^20^ These locally driven efforts can help build momentum for a wider health system change. By fostering trust, empowering communities and aligning care with people’s values, health systems can move towards whole health. Lasting change for people and communities occurs when health is co-created, not prescribed.
